# The evaluation of the impact of titania nanotube covers morphology and crystal phase on their biological properties

**DOI:** 10.1007/s10856-015-5495-2

**Published:** 2015-03-20

**Authors:** Żaneta Lewandowska, Piotr Piszczek, Aleksandra Radtke, Tomasz Jędrzejewski, Wiesław Kozak, Beata Sadowska

**Affiliations:** 1 Department of Inorganic and Coordination Chemistry, Faculty of Chemistry, Nicolaus Copernicus University, ul. Gagarina 7, 87-100 Toruń, Poland; 2 Department of Immunology, Faculty of Biology and Environment Protection, Nicolaus Copernicus University, ul. Lwowska 1, 87-100 Toruń, Poland; 3 Department of Infectious Biology, Faculty of Biology and Environmental Protection, University of Lodz, ul. S. Banacha 12/16, 90-237 Łódź, Poland

## Abstract

**Abstract:**

The highly ordered titanium dioxide nanotube coatings were produced under various electrochemical conditions on the surface of titanium foil. The anodization voltage changes proved to be a main factor which directly affects the nanotube morphology, structure, and wettability. Moreover we have noticed a significant dependence between the size and crystallinity of TiO_2_ layers and the adhesion/proliferation of fibroblasts and antimicrobial properties. Cellular functionality were investigated for up to 3 days in culture using a cell viability assay and scanning electron microscopy. In general, results of our studies revealed that fibroblasts adhesion, proliferation, and differentiation on the titania nanotube coatings is clearly higher than on the surface of the pure titanium foil. The formation of crystallic islands in the nanotubes structure induced a significant acceleration in the growth rate of fibroblasts cells by as much as ~200 %. Additionally, some types of TiO_2_ layers revealed the ability to the reduce of the staphylococcal aggregates/biofilm formation. The nanotube coatings formed during the anodization process using the voltage 4 V proved to be the stronger *S. aureus* aggregates/biofilm inhibitor in comparison to the uncovered titanium substrate. That accelerated eukaryotic cell growth and anti-biofilm activity is believed to be advantageous for faster cure of dental and orthopaedic patients, and also for a variety of biomedical diagnostic and therapeutic applications.

**Graphical Abstract:**

The highly ordered titanium dioxide nanotube coatings were produced under various electrochemical conditions on the surface of titanium foil. The anodization voltage changes proved to be a main factor which directly affects the nanotube morphology, structure, and wettability. Moreover we have noticed a significant dependence between the size and crystallinity of TiO_2_ layers and the adhesion/proliferation of fibroblasts and antimicrobial properties.
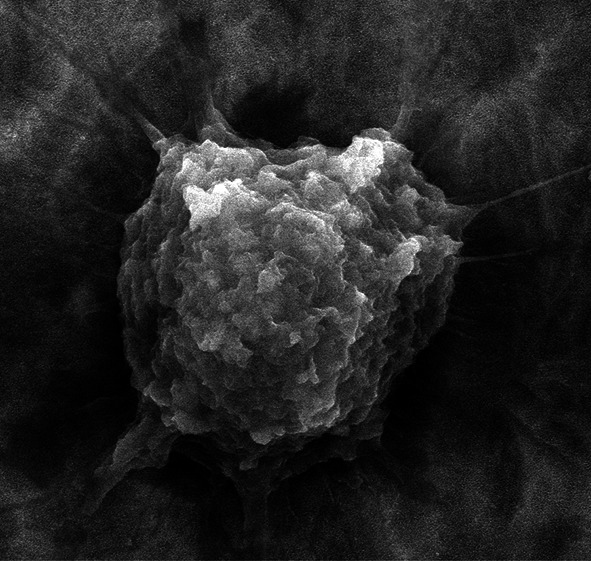

## Introduction

Over the past decades, titanium and its alloys have found widely application as the biomaterials used for implants development. Those materials exhibit appropriate properties like good biocompatibility and mechanical characteristic, corrosion resistance, and processability [1–3]. However, pure titanium is a kind of bioinert material and could not be so easily biologically integrate with the bone tissue and also did not acquire bactericidal capabilities. It is well known that different interactions occur between the surface of biomaterials and the biological environment after they implantation into the recipient organisms [4–7]. The first stage is interaction with body fluids and ions, then extracellular matrix proteins (ECM) non-specific adsorption occurs. Afterwards, neutrophils and macrophages interrogate the implant. The phagocytes activation and cytokines released are considered to attract fibroblasts and lead the foreign body covering process. Biocompatibility of artificial implants is largely determined by their surface characteristics such as surface morphology, microstructure, composition, and properties [8]. The formation of titanium dioxide nanotube layers (TNT) on titanium surface is one of the methods to stimulate bone growth and osteogenic cells to improve the osteointegration [9]. A considerable number of studies have proved that the titania nanotubes has the following advantages: (a) the nanotubular structures increase significantly the surface area and favors to: eukaryotic cells deposition, (b) they may serve as drug delivery system because their length and diameter can be controlled precisely, and (c) nanotube surface is created directly from the underlying native titanium alloys, and it has the same component as occurs in the alloys [10–16].

Many research groups reported that TiO_2_ nanotubes can modulate the functions of many cell types, such as fibroblasts, osteoblasts, mesenchymal stem cells, and endothelial cells [17–19]. In our work, we focused on the production of TiO_2_ nanotubes by the anodic oxidation of titanium foil at various voltage and studies on the dependency between morphology and structure of nanotubes formed and their biological (immunological and antimicrobial) properties. This problem is very important for the development of a new generation of implants used for example in facial surgery. In the present paper, the results of our investigations of the above-mentioned dependencies on TiO_2_ nanotube coatings wettability, the adhesion/proliferation of fibroblasts and antimicrobial properties are discussed. Moreover, we demonstrate that the surface morphology and crystallinity of the TNT covers can be well controlled by varying oxidization conditions, which consequently brings important property changes to the biomaterials.

## Materials and methods

### Synthesis and characterization of TiO_2_ nanotube coatings

TiO_2_ nanotube layers (TNT) were produced on the surface of 5 × 50 mm titanium foil pieces (Aldrich, 99.7 % purity) as a result of the anodic oxidation. Before the anodization process, foil pieces were cleaned by ultrasonication for 15 min in acetone, ethanol, distilled water, and dried in Ar stream. The pre-treated samples were then chemically etched in the 1:4:5 mixture of HF, HNO_3_, and distilled water by 30 s. Finally, the titanium substrate was washed with distilled water and dried in Ar stream. The electrochemical experiments were carried out at room temperature using the electrolytic reactor that was composed of the titanium foil (anode) and platinum wire (cathode) immersed in an electrolyte solution (0.3 wt% aqueous HF solution). The distance between electrodes was ~2 cm. The anodization voltage (*U*) was varied from 3 V up to 20 V, using the anodization time t = 20 min. In order to purify, the produced coatings were washed with distilled water with the addition of Al_2_O_3_ powder (averaged particle size = 50 nm) in an ultrasonic bath for 1 min, and then dried in Ar stream.

Morphology of produced coatings were studied using Quanta field-emission gun scanning electron microscope (Schottky FEG). A 30.0 kV accelerating voltage was chosen for SEM analysis and the micrographs were recorded under high vacuum using secondary electron detector (SE). The conventional micrographs were taken with transmission electron microscope (TEM; Tecnai F20 X-Twin). Structural studies of the produced TiO_2_ nanotube layers were performed using a high-resolution transmission electron microscopy (FEI type TECNAI G2TF20-X-TWIN) and the diffuse reflectance infrared Fourier transform spectroscopy (DRIFT; PerkinElmer Spectrum 2000 spectrophotometer). All reflectance data was transformed to Kubelka–Munk units (K–M) and presented as a function of wave number. The wetting angles of TNT coatings were investigated using a Krüss system for the wetting angle measurement. The contact angle from ~15 µl of distilled water sessile droplets (0, 30, 60, 90, 120, 150, 180 s after being dropped on the surface) was measured for selected samples. The value of contact angle for each biomaterial was the average value of ten measurements.

### Cell culture condition

Murine fibroblasts cell line L929 (American Type Culture Collection) were cultured at 37 °C in 5 % CO_2_ and 100 % humidity in complete RPMI 1640 medium containing 2 mM l-glutamine (Sigma-Aldrich, Germany), 10 % heat-inactivated fetal bovine serum (FBS), 100 mg streptomycin/ml, and 100 IU penicillin/ml (PAA Laboratories GmbH, Germany). L929 cells were grown in 25 cm^2^ cell culture flasks (Corning, USA) and the culture medium was changed every 2–3 days. The cells were passaged when reaching 70–80 % confluency.

### Cell adhesion and viability (after 24 h) and proliferation (after 72 h)

The effects of TiO_2_ nanotubes on the cell adhesion (after 24 h) and proliferation (after 72 h) were assessed using the MTT assay. The cells at a density of 1 × 10^4^ cells/well (in 1 ml of complete RPMI 1640 medium) were incubated with sterilized biomaterials in a 24-well culture plates for 24 and 72 h. After the respective co-incubation time, biomaterial samples were washed with phosphate buffered saline (PBS) three times and transferred to a new 24-well plate. Then, 500 μl of culture medium (RPMI 1640 without phenol red; Sigma-Aldrich, Germany) and 500 μl of MTT (5 mg/ml in PBS prepared and filtered immediately before use) were added to each well. After 3 h of incubation at 37 °C in 5 % CO_2_, the medium was replaced with 500 μl of dimethyl sulfoxide (100 % v/v; Sigma-Aldrich, Germany). The plate was shaken for 10 min, and then the solution in each well was transferred to a 96-well ELISA plate. The absorbance of the solution was measured at a wavelength of 570 nm with the subtraction of the 630 nm background using a plate reader (Synergy HT; BioTek, USA). The blank groups (TNT incubated without cells) were treated with the same procedures as experimental groups. Culture medium without the TNT served as a negative control in each experiment. All measurements were done in duplicate in three independent experiments.

### Bacterial strains and aggregates/biofilm formation on titanium biomaterials

*Staphylococcus aureus* ATCC 29213 reference strain and *S. aureus* H9 clinical strain were grown for 24 h at 37 °C on Müeller-Hinton Agar (MHA; BTL, Poland). Then, single colony was transferred to 5 ml Tryptical Soy Broth (TSB; BTL, Poland) containing 0.25 % glucose (TSB/Glu) and incubated for the next 18 h at 37 °C. Microbial suspensions at the optical density OD_535_ = 0.9 (nephelometer type Densilameter II, Czech Republic), corresponding to 5 × 10^7^ CFU/ml, were prepared and added (1 ml) to the wells of a 24-well tissue culture polystyrene microplate (Nunc, Denmark). Then, the pieces (about 5 × 5 mm) of titanium foil covered with the titanium dioxide nanotubes (biomaterials tested) and without such coating (control K1) were placed into *S. aureus* suspensions for 24 h and incubated in stable conditions at 37 °C to form microbial aggregates/biofilm. Staphylococcal culture alone (without biomaterial) was used as a microbial growth positive control (K+). Negative control wells consisted of biomaterial tested in TSB/Glu (control K2) and TSB/Glu only (K−).

### The assessment of *S. aureus* aggregates/biofilm on titanium foil

To evaluate aggregates/biofilm formation, LIVE/DEAD BacLight Bacterial Viability kit (L/D; Molecular Probes, USA), Alamar Blue (AB; BioSource, USA) staining and CFU method were used. For all types of titanium foil modified, two independent sets of experiments were prepared, each in quadruplicate. Bacterial cells weakly bounded with the surface of biomaterials were gently removed and the pieces of titanium foil were vortexed (3 min) to reclaim the bacteria forming aggregates/biofilm on them. Obtained *S. aureus* suspensions or TSB/Glu (negative controls) were added (100 μl) to the wells of 96-well tissue culture polystyrene plates (Nunclon Surface, Nunc, Denmark) in quadruplicate to apply L/D and AB test. For L/D staining a mixture of Syto9 and propidium iodide (PI) in tissue culture water (Sigma, Germany) was prepared as recommended by the manufacturer. Then, bacterial suspensions and medium (negative control) were stained with the mixture of dyes (100 μl/well) for 20 min at room temperature in the dark. Finally, the fluorescence of the wells (at 485ex/535em nm for green Syto9 and at 485ex/620em nm for red PI) was measured. The results were presented as percentage of live bacteria reclaimed from aggregates/biofilm formed on titanium foils tested, calculated from the mean fluorescence values ± standard deviation (SD) of the control K1 (considered as 100 %) and test wells. Staining protocol for AB was used as recommended by the manufacturer by adding 5 µl of Alamar Blue to the wells of 96-well tissue culture polystyrene plates containing bacterial suspensions and medium (negative control). Then, the plates were incubated for 1 h at 37 °C (with shaking). Finally, the absorbance was determined at 550 and 600 nm using a multifunctional counter (Victor2; Wallac, Finland). The percentage of AB reduction was calculated according to the manufacturer formula:

$$AB_{{\text{Re} duction}} [\% ] = \frac{{\varepsilon_{OX} \lambda_{2} \cdot A\lambda_{1} - \varepsilon_{OX} \lambda_{1} \cdot A\lambda_{2} }}{{\varepsilon_{RED} \lambda_{1} \cdot A'\lambda_{2} - \varepsilon_{RED} \lambda_{2} \cdot A'\lambda_{1} }} \cdot 100$$

where *ε*_OX_—molar extinction coefficient of AB oxidized form, *ε*_RED_—molar extinction coefficient of AB reduced form, *A*—absorbance of test wells, *A’*—absorbance of negative control well *λ*_1_—550 nm, *λ*_2_—600 nm.

Obtained results were presented as percentage of metabolically active bacteria reclaimed from aggregates/biofilm formed on titanium foils tested, calculated from the mean percentage of AB reduction ± SD of the control K1 (considered as 100 %) and test wells. Parallelly, bacterial suspensions tested were diluted from 10^−1^ to 10^−5^ in phosphate buffered saline (PBS; Biomed, Poland) preceded by intensive vortexing. Then 100 μl of staphylococcal suspensions (10^−4^–10^−5^) was cultured on MHA and colony forming units (CFU) were counted after 24 h incubation at 37 °C. The experiment was performed twice and each bacterial culture was prepared in duplicate. The density of initial staphylococcal suspensions was calculated using the average value of CFU counts.

### Preparation of sample for SEM analyses

SEM analyses were performed to study the morphology of fibroblasts grown on the surface of both control and anodized titanium plates. For these purpose, after cell culture for 24 and 72 h, biomaterial samples were washed three times with phosphate-buffered saline (PBS) for 10 min each wash to remove the non-adherent cells and the cell culture medium. The cells were then fixed using freshly prepared 2.5 % glutaraldehyde in distilled water at 4 °C for minimum 4 h (maximum 1 week). After removal of the glutaraldehyde solution, the samples were sequentially dehydrated in a graded series of ethanol (50, 70, 90, and 100 %) for 10 min first three and the last one for 20 min. Finally, the samples were dried in vacuum-assisted desiccators overnight and then stored at room temperature till SEM analysis was carried out.

### Statistical analysis

The results were reported as mean ± standard error mean (*S*.*E*.*M*.) and were analyzed by one-way ANOVA followed by the post hoc paired Student’s two-tailed *t* test or by *U* Mann–Whitney test for Statistical 6.0. The level of significance set at *P* < 0.05. The significance levels were marked in the figures as: *, if *P* < 0.05, **, if *P* < 0.01, and ***, if *P* < 0.001).

## Results and discussion

### Production and characterization of nanomodified surfaces

**Fig. 1:**
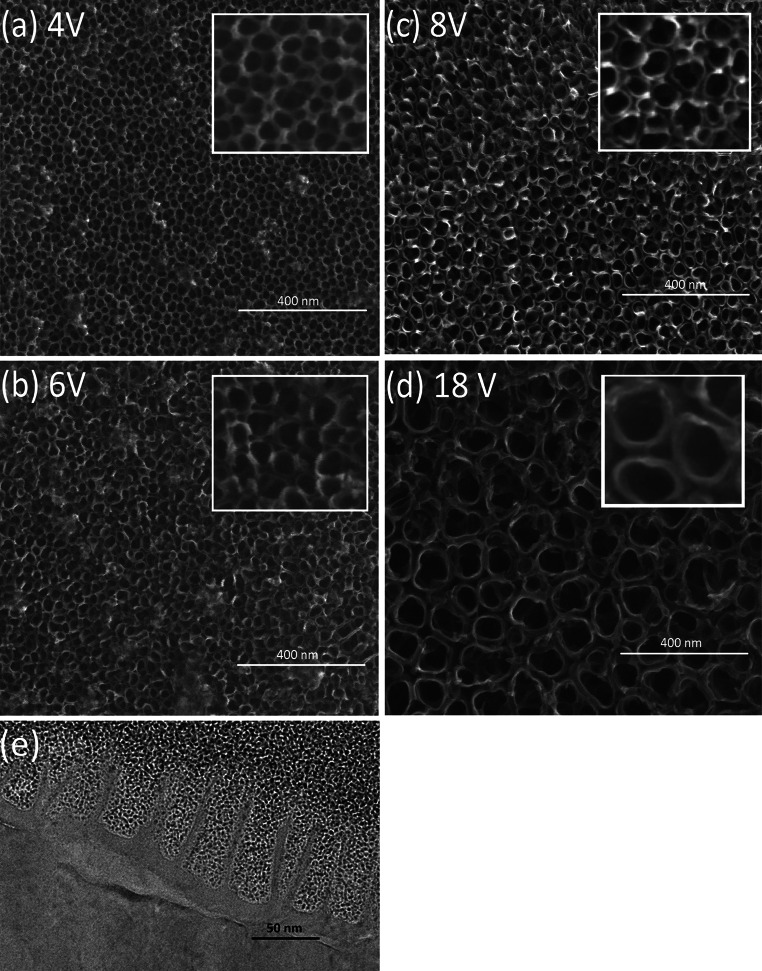
SEM images of titanium foil anodized at different applied voltage, varying from 4 V up to 15 V (**a**–**d**), and cross-sectional TEM image of the nanotubes (**e**)

**Fig. 2:**
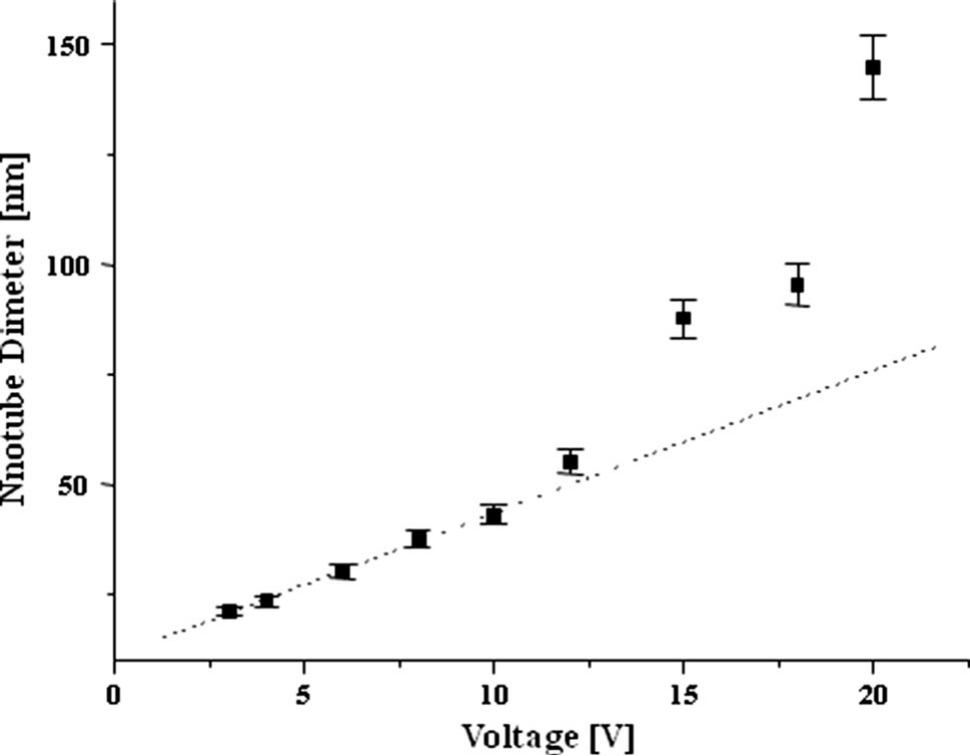
The relationship of the nanotube diameters versus voltage, estimated basing SEM data

**Fig. 3:**
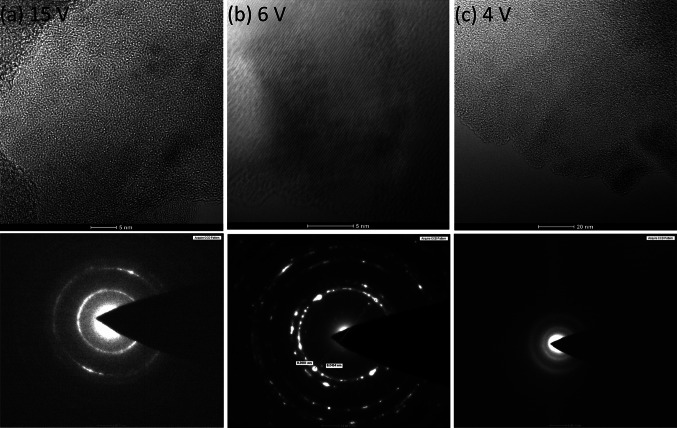
TEM micrographs of titania nanotubes obtained using different voltage: **a** 15 V (TNT 15), **b** 6 V (TNT 6), and **c** 4 V (TNT 4) respectively

Titanium dioxide nanotube (TNT) coatings were produced on the surface of titanium foil (99.7 % purity) leading the anodizing process at room temperature for t = 20 min in the range of 3–20 V. A current density within the scope of 5.0–23.8 mA/cm^2^ was sufficient for the whole range of applied potential. Analysis of SEM and TEM images revealed that formed nanotubes were open at the top, closed at the bottom, and their diameter and shape significantly depend of the applied potential (Fig. 1). The diameters of tubes (estimated basing SEM data and the color of the coating [20]) produced at low potentials, i.e. between 3 and 5 V (TNT 3–TNT 5), were about 20–30 nm. Moreover, analysis of SEM images revealed that formed tubes were connected by common walls (Fig. 1a). The effect of the nanotubes separation has been initiated at 6 V (TNT 6, Fig. 1b), and at higher potentials (8–20 V) the formation of TNT coatings composed of separated nanotubes with diameters of 50–145 nm (TNT 8–TNT 20) was observed (Figs. 1c, d, 2). Analysis of SEM data revealed that the diameter of nanotubes increased proportionally to the applied voltage only between 3 and 8 V (Fig. 2). The use of higher potentials (8–20 V) caused the rapid increase of nanotubes diameter and the increase of TNT coatings thickness. Appearances of this effect have been also noted in earlier reports [21, 22], but the exact formation mechanism is not fully understood. It is known that it is a direct consequence of competition between the chemical dissolution rate of TiO_2_ by fluoride ions and the electrochemical formation rate, which includes both the oxidation of Ti and electric field induced etching of TiO_2_ [13, 23]. The structure of produced TNT coatings is one of the important factors which can influence the mechanism of their formation and biological properties. The previous reports exhibited that anodized TiO_2_ nanotubes are amorphous in nature and can be converted into anatase, rutile or a mixture of these polycrystalline forms by annealing above 280 °C [24–30]. Analysis of HRTEM images and SAD patterns revealed that the amorphous coatings were formed only for the lower applied voltages; *U* < 5 V (Fig. 3). Studies of the TNT 4 diffuse reflectance FTIR spectrum (DRIFT), revealed the presence of bands at 718, 772, and 940 cm^−1^, which were assigned to Ti–O vibrations of TiO_4_ tetrahedrons. This confirms the amorphous structure of TNT coatings produced at lower potentials [20, 31, 32]. HRTEM studies proved that crystalline nanotube layers were formed about *U* = 6 V (TNT 6, Fig. 3). The appearance of bands at 868 and 710 cm^−1^, in the DRIFT spectrum of TNT 6 suggests that structure of this sample consists of TiO_2_ nanocrystals, e.g. anatase or anatase and rutile mixture [20, 31, 32]. The amorphous coatings containing anatase grains were produced at higher voltage (*U* = 8–20 V), what was confirmed by analysis of HRTEM (TNT 15, Fig. 3) and DRIFT data.

**Fig. 4:**
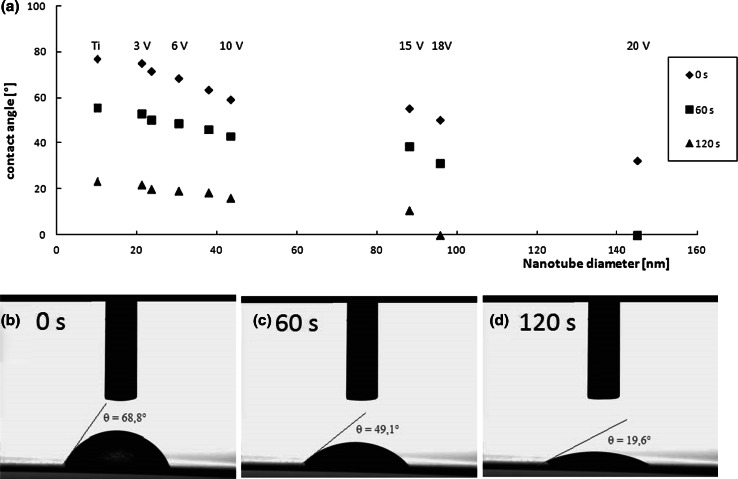
The contact angle behavior on Ti and TiO_2_ nanotube layers (**a**), and optical images of water droplets on TiO_2_ nanotubes with diameters 30 nm, measurements made after **(b)** 0 s, **(c)** 60 s, and (**d**) 120 s

Surface wettability is one of the most important parameters affecting biological response to implanted biomaterials, which affects protein adsorption, platelet adhesion/activation, blood coagulation and cell adhesion. [33, 34] As presented at Fig. 4 the contact angle for all samples of titania nanotubes were lower than for pure Ti foil, for which contact angle equals 77.2°. Additionally, with increase of the nanotube diameter the contact angles become lower, which means that nanotube coatings become much hydrophilic. Wetting behavior of nanotubes with smaller diameter, i.e. 20–30 nm (3–5 V) did not differ significantly from those for pure titanium. The differences of contact angles, registered for TNT 3 and TNT 4 were lower than 10° in comparison to the reference sample (Fig. 4). Increasing of hydrophilic properties occurred for TNT coatings with diameter of tubes higher than 30 nm, the value of contact angle decreased even to the 32.7° for the TNT 20 with diameter around 145 nm (20 V).

### Fibroblasts adhesion and proliferation

**Fig. 5:**
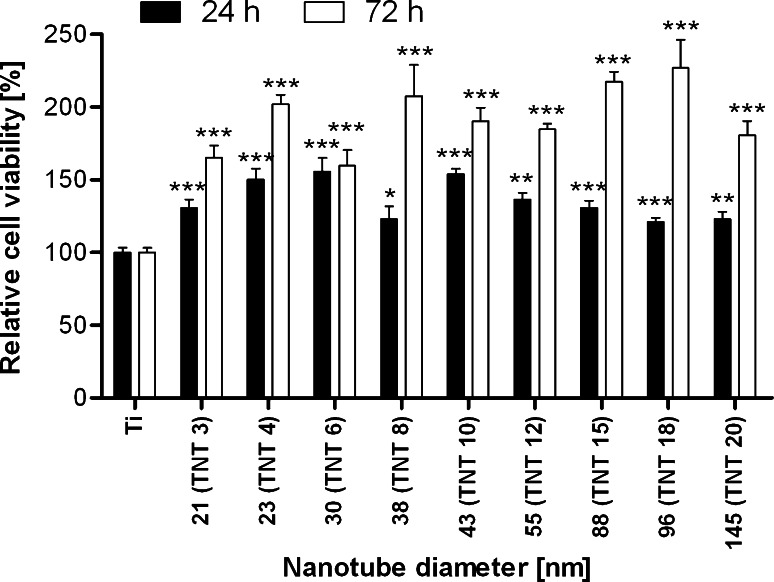
Effect of TiO_2_ nanotubes diameters on murine fibroblasts L929 adhesion (after 24 h) and proliferation (after 72 h) on the nanotubes surface detected by MTT assay cell viability. Cell viability was assessed by an MTT assay for 24 h and 72 h incubation with the respective nanotubes. Data are expressed relative to the of control cells that were treated identically, but only with pure Ti plates (=100 %). Values are expressed as mean ± S.E.M. of three experiments. *Asterisks* indicate significant difference between the fibroblasts incubated with TiO_2_ nanotubes in comparison to the cells incubated with pure Ti plates in respective incubation time (24 h-*black columns*, 72 h-*white columns*; **P < 0.01, *P < 0.001)

**Fig. 6:**
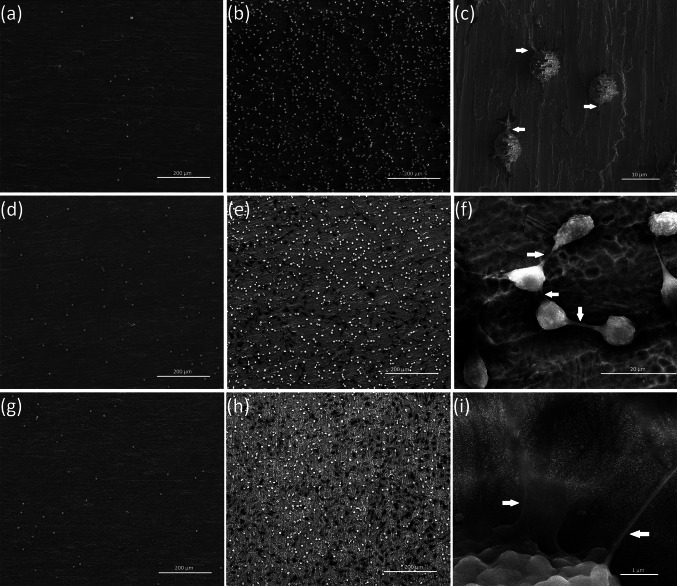
SEM images showing the cell adhesion (24 h) and proliferation (72 h) of L929 murine fibroblasts on the pure Ti plates and TiO_2_ nanotubes with the diameters of 30.2 and 87.8 nm after 24-h (**a**, **d**, **g**) and 72-h (**b**, **e**, **h**) incubation time, respectively. The *arrows* in the figure (**c**, **f**, **i**) indicates the filopodia spread between fibroblast cells

The effect of different morphology and phase structure of TiO_2_ nanotubes on the L929 murine fibroblasts adhesion (after 24 h) and proliferation (after 72 h) were examined using MTT assay, as shown in Fig. 5. In order to facilitate interpretation of the data the following symbols were introduced: 3 V—TNT 3, 4 V—TNT 4, on the same basis the rest of the samples were rewritten. With an increase of incubation time, more cells adhered on the all samples, except nanotubes obtained at voltage 6 V (TNT 6) with a diameter of 30 nm (*P* = 0.79). This may be related to the appearance of order crystalline structure, which has not been fully created. Probably, these conditions were not quite suitable for adhesion and proliferation of the cells. Moreover, the number of cells on TNT surface was significantly higher in comparison to the cells adhered to pure Ti plates, which was expressed as a 100 %. The results also indicated that the increasing diameter of TNT led to greater proliferation of fibroblasts on their surface, which was observed after 72-h incubation time. Increase in the cell proliferation was noticed, particularly in nanotubes with diameter of: 23 nm (TNT 4), 38 nm (TNT 8), 88 nm (TNT 15) and 96 nm (TNT 18). This phenomenon was not observed, however, after 24-h incubation time. Similarly, Yu et al. [35] showed that the proliferation rates of cells cultured on TNT layers enhanced with increasing tube diameter from 20 to 145 nm, which may be attributed to different length and nanometer-scale roughness of the nanotube layers. Our results also showed that the fibroblasts adhesion and proliferation on TNT are significantly higher, especially after 72 h culture by as much as 60–130 % as compared to the pure Ti surface. These findings correspond with the results from SEM analysis (Fig. 6), which showed that the number of cells on the surface of TNT was significantly greater after 72-h incubation time compared to the 24-h incubation, especially in relation to the number of fibroblasts observed on control incubation with pure Ti plates. Similarly, the other researchers also demonstrated the significant differences in the adhesion and proliferation of osteoblasts and fibroblasts incubated with pure Ti and TiO_2_ nanotubes [36–38].

### Morphology of adherent fibroblasts

On Fig. 6 comparative SEM micrographs of L929 murine fibroblasts cultured on the pure Ti and TiO_2_ nanotubes with the diameters of 30 (TNT 6) and 88 nm (TNT 15) for 24-h (a–c) and 72-h (d–f) were shown, respectively. Regarding the examination by SEM, the cells cultivated on the nanotubes surface effectively attached to the plates surface. Importantly, the fibroblasts cultured on pure titanium surface still remained in their primary (non-adherent) round shape, while the cells incubated on TNT attached to the surface and started to form filopodia (arrows in Fig. 6c, f, i). Studies using several cell types have shown that filopodia are sensitive to nanotopography of attached surface resulting in the changes of cell morphology by filopodial guidance. [39, 40] Recently, Swan et al. [41] described filopodia extending progresses into the nanopores, which increased the osteoblast adhesion. Oh et al. [36] and Popat et al. [42] reported that the filopodia of cell extensions were protruding into the nanotubular architecture using different cell types. According to these research findings, it is possible to consider that the TNT may constitute the attractant for the filopodia facilitating fibroblasts attachment (arrows in Fig. 6c, f, i). Moreover, our data from the MTT assays showed that the TNT facilitates adhesion (24 h incubation times) and proliferation (72 h incubation times) of fibroblasts (Fig. 5).

Because of the fact, that a favorable cellular interaction with biomaterial surface is critical for the success of long-term implantation [43], our results can suggest the biocompatible properties of the tested nanotubes. However, further researches are required to verify the impact of TiO_2_ nanotubes on the viability and proliferation of the other cells, such as osteoblasts. A better understanding of osteogenic cells interaction with TNT in vitro can help to design the implants more suitable for bone regeneration in vivo. For orthopedic implant success, both osteoblasts and fibroblasts play a pivotal role in the osteointegration and wounds healing [44].

### Antimicrobial activity

**Fig. 7:**
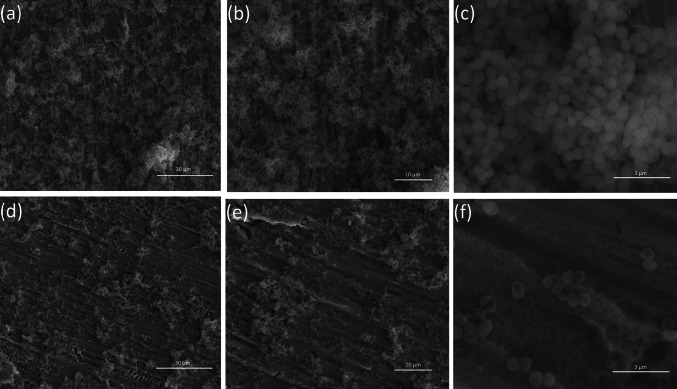
SEM micrographs of *S. aureus* on uncovered titanium foil—K1 (**a**, **b**, **c**) and titanium dioxide nanotubes obtained at 4 V—TNT 4 (**d**, **e**, **f**)

**Fig. 8:**
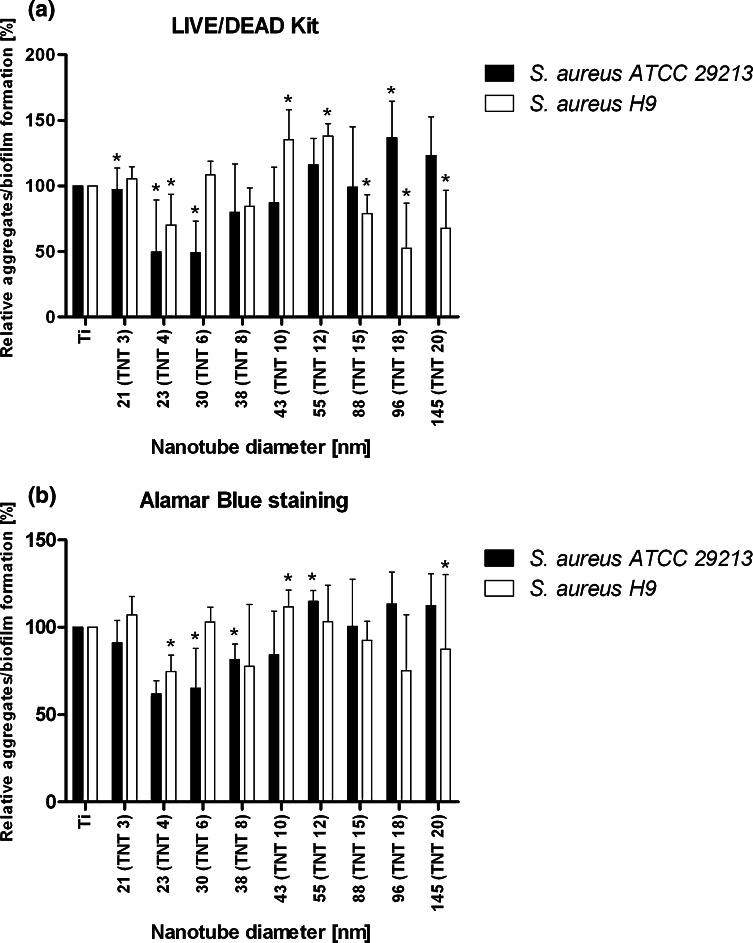
*S. aureus* aggregates/biofilm formation on the surfaces of titanium foil covered with the TiO_2_ nanotubes tested by LIVE/DEAD BacLight Bacterial Viability kit and Alamar blue staining. The results are presented as mean percentage ± standard deviation (SD) of live bacteria reclaimed after 24 h from nanotubular TiO_2_ surfaces compared to conventional titanium foil (considered as 100 %). Two independent sets of experiments were prepared, each in quadruplicate. *Significant differences, *P* < 0.05 in both independent sets of experiments

Both *S. aureus* strains were able to form aggregates/biofilm on the surface of all titanium biomaterials tested (Figs. 7, 8). However, we demonstrated that some types of titanium dioxide nanotube covers reduced staphylococcal aggregates/biofilm formation. As it was shown in Fig. 8, biomaterial marked TNT 4 (titanium foil anodized at a potential 4 V) was the most potent *S. aureus* aggregates/biofilm inhibitor. Observed effect was not spectacular, however TNT 4 caused the limitation of bacterial aggregates/biofilm formation, in comparison to uncovered titanium foil (K1), achieving for *S. aureus* ATCC 29213 50.4 ± 39.8 % (*P* < *0,05*) and 38.2 ± 7.6 % (*P* < 0.001) tested by L/D and AB staining, respectively, and for *S. aureus* H9 reaching, similarly, 29.7 ± 23.2 and 25.5 ± 9.5 % (*P* < 0.05). Interestingly, TNT 6 and TNT 18 exhibited similar anti-biofilm activity, but targeted against only one of staphylococcal strains tested. *S. aureus* ATCC 29213 aggregates/biofilm biomass deposited on TNT 6 decreased by 51.1 ± 24.1 and 34.9 ± 22.7 % (*P* < 0.05) tested by L/D and AB staining, respectively, compared to pure Ti plates. While TNT 18 was potent aggregates/biofilm inhibitor for *S. aureus* H9 causing 47.4 ± 34.1 % (L/D staining; *P* < 0.05) and 24.8 ± 32.0 % (AB staining; *P* < 0.05 for set 1 and *P* = 0.665 for set 2) inhibition of aggregates/biofilm formation.

The positive effect of anodized titanium substrates on implant tissue-integration is well-documented, however their antimicrobial activity, including anti-adhesive and anti-biofilm properties are controversial. From the one side, we can expect preferential microbial adhesion to modified biomaterials as a result of greater roughness of nanostructured surfaces compared with pure (smooth) titanium. Such observation was made by Puckett et al. [45] in case of *S. aureus*, *S. epidermidis* and *Pseudomonas aeruginosa* adhesion to the anodized nanotubular and nanotextured titanium surfaces. On the other hand, the inhibition of mentioned above bacteria adherence to nanorough titanium biomaterials created by electron beam evaporation was demonstrated [45, 46]. Ercan et al. [47] observed a significant decrease in *S. aureus* biofilm formation on anodized nanotubular titanium, but after 2 days culture. While, the initial adherence of staphylococci (1–8 h) on nanotubular surfaces was stronger in comparison to conventional titanium. It has been suggested that the presence of fluorine on the anodised Ti surfaces could explain such anti-biofilm effect [45, 47]. Our results showing strictly defined titanium dioxide nanotubes (TNT) covers as microbial biofilm formation inhibitors indicate the effect of their physical structures (nanotopography) rather than chemical composition. Even if antimicrobial properties of biomaterial tested was associated with the presence of fluorine on the top layer of the anodized nanotubular surfaces after using HF as an electrolyte during the anodization process as was described earlier [45, 47], all TNT cover types tested should possess similar activity. Whereas, it is known that anodic oxidation performed under increasing voltage led to obtain the nanotubes with different diameters and various physical form (amorphous or crystalline). Both parameters, together with chemistry and hydrophilicity, were considered important in biomaterial response to bacteria including *S. aureus* and *S. epidermidis* [48]. In our study the form of TNT coatings was dependent on applied voltages. Typical amorphous coatings developed under *U* < 5 V, while at higher voltage crystalline grains were produced. The most notable activity of all surfaces tested against *S. aureus* aggregates/biofilm formation (irrespective of the strain used) was observed for TNT 4 possessing amorphous structure and nanotubes with diameter about 20–30 nm. Similarly Ercan et al. [48] reported reduced number of live staphylococci adhering to the surface of nanotube with a diameter of 30–80 nm. Interestingly, Cendrowki et al. [49] noticed strong antibacterial activity (against *Escherichia coli*) of nanocrystallized titanium covered on silica nanotubes. However, that effect was observed after surface irradiation with the artificial visible and ultraviolet light and was based on a well-known photocatalytic activity of titania in anatase structure. Also other researchers reported decreased microbial adhesion to crystalline (anatase or rutile) TiO_2_ even without light-stimulation [45, 48]. This could explain anti-biofilm activity of TNT 6 and TNT 18 containing nanocrystals of the anatase form or anatase and rutile mixture as was revealed from DRIFT analysis. This reflects only the dependence of antimicrobial properties of biomaterial tested on applied *S. aureus* strain.

Problems concerning the modification of titanium surfaces in order to develop more biocompatible biomaterials with antimicrobial activity of implants are especially widely studied in recent years. Zhang et al. [50] used titanium surfaces modified by nanotubes containing vancomycin and obtained approximately 40 % decrease in *S. aureus* adhesion. Lin et al. [51] showed significant limitation of *S. aureus* and *S. epidermidis* adhesion and biofilm formation by gentamicin-loaded titania nanotubes. In this light, our results on the inhibition of staphylococcal biofilm formation by selected TNT covers without the use of antibiotics seems to be promising. Antibacterial activity of titanium coated by hydroxyapatite without the assistance of pharmaceuticals was also observed by Mathew et al. [52]. Nevertheless, further preclinical and clinical studies are required, since implant based on anti-adhesive for bacteria biomaterials can still develop infections in vivo as it was noticed by Zhang et al. [50].

## Conclusions

Results of our investigations of electrochemical anodization processes in the potential range 3–20 V revealed two growth mechanisms of nanotubes (TNT) on surface of titanium foil. A linear dependence between usage voltage and TNT diameter was observed in the range 3–12 V, while for higher potentials a positive deviation from linearity has been noticed. Analysis of HRTEM and DRIFT data showed that the amorphous coatings have been formed below 5 V. Whereas between 8 and 20 V, the amorphous TNT layers containing anatase grains were produced. It’s interesting that TNT layers produced at 6 V revealed the nanocrystalline structure.

Cellular functionality in terms of adhesion, proliferation, cytoskeletal organization and morphology were investigated for up to 3 days culture using MTT assay and scanning electron microscopy. The number of cells adhering to the TNT surface was superior than that on the Ti polished, which is most likely due to the more differentiated topological features and significantly higher surface area. The only exceptions of this rule are TNT samples produced at 6 V, which may be associated with the appearance of large amount of crystalline grains, e.g. anatase or anatase and rutile mixture. In addition, cell proliferation was found to be dependent of nanotube diameter and phase structure, which are closely linked to the value of used voltage. Importantly, the fibroblasts incubated on TiO_2_ nanotubes attached to the surface and started to form filopodia, which actually were going into the nanotubes pores, producing an interlocked cell structure.

Furthermore, extensive works have been conducted on the TNT antibacterial activity against *S. aureus*. It has been demonstrated that some types of titanium dioxide nanotube covers were able to reduced staphylococcal aggregates/biofilm formation. The best results have been obtained for the TNT samples produced at 4 V. Both behaviors, immunological and microbiological activity, are extremely important in use as bioactive surface layer for orthopedic and dental applications. From the point of view of the structure of implants surface, the most promising are coatings obtained with 4 and 18 V. The finding of this work, may contribute significantly to the new type of efficient biocompatible implant materials.
